# Psychocardiology in a heartbeat: cardiac complications to consider in psychopharmacology

**DOI:** 10.1192/j.eurpsy.2022.1863

**Published:** 2022-09-01

**Authors:** M. Conde Moreno, F. Ramalheira, R. Amador

**Affiliations:** 1 Centro hospitalar Psiquiátrico de Lisboa, Hospital De Dia, Lisboa, Portugal; 2 Centro hospitalar Psiquiátrico de Lisboa, Serviço De Electroconvulsoterapia, Lisboa, Portugal; 3 Centro Hospitalar Lisboa Ocidental, Cardiologia, Carnaxide, Portugal

**Keywords:** Antidepressants, Antipsychotics, Cardiology, Complications

## Abstract

**Introduction:**

Antidepressants and antipsychotics have a wide range of cardiac side effects. Although the absolute risk is considered low, some are potentially life-threatening.

**Objectives:**

We aim to review the main cardiological complications of antidepressants and antipsychotics and their management. We will consider 1) QTc prolongation and arrhythmia 2) heart rate 3) blood pressure 4) myocarditis.

**Methods:**

Review of cardiological complications of antidepressants and antipsychotics.

**Results:**

Qtc prolongation is correlated with arrhythmia risk. QTc is obtained with Bazett’s formula, which has limitations. All inpatients and some outpatients starting antipsychotic should undergo ECG. Increased QTc can result in different approaches, depending on severity. Most antidepressants do not significantly affect QTc, except for escitalopram and tricyclics, mostly in overdose. Sinus tachycardia can occur with most antipsychotics. Tricyclics can also produce this effect. Other causes should be excluded, and management can be achieved with bisoprolol. Other antidepressants most commonly produce a slight decrease in heart rate or have a minimal to no effect. Antipsychotics can cause hypertension or hypotension depending on the degree of affinity to specific adrenergic receptors. Tricyclics can lead to postural hypotension. Antidepressants interfering with noradrenaline can cause hypertension. Myocarditis is mostly associated with clozapine. Patients should be screened for clinical signs and laboratory findings - especially in the presence of risk factors. Suspicion should prompt echocardiological examination and confirmation leads to cardiology referral.

**Conclusions:**

Weighing the risks and benefits of these medications is a continuous process. Management of cardiological complications is possible and may involve a multidisciplinary approach.

**Disclosure:**

No significant relationships.

